# Defining Reference Intervals for Complete Blood Count and Micronutrient Parameters in Urban Bangladeshi Population

**DOI:** 10.3390/biom16070968

**Published:** 2026-06-30

**Authors:** Md. Ahsanul Haq, Kiyoshi Ichihara, Dewan Zubaer Islam, Md. Jakarea, Mohammad Mehedi Hasan, Anjan Kumar Roy, Evana Akhtar, Rubhana Raqib, Protim Sarker

**Affiliations:** 1 Immunobiology, Nutrition and Toxicology, Nutrition Research Division, International Centre for Diarrhoeal Disease Research, Bangladesh (icddr,b), Dhaka 1212, Bangladesh; shohag@icddrb.org (M.A.H.); dewanzubaerislam@gmail.com (D.Z.I.); md.jakarea@icddrb.org (M.J.); mohammad.hasan1@icddrb.org (M.M.H.); anjan@icddrb.org (A.K.R.); evana@icddrb.org (E.A.); rubhana@icddrb.org (R.R.); 2 Department of Clinical Laboratory Science, Faculty of Health Sciences, Yamaguchi University Graduate School of Medicine, Ube 755-8505, Japan; ichihara@yamaguchi-u.ac.jp

**Keywords:** CBC, LAVE, reference intervals, RI, micronutrient, hematological, vitamin D, Bangladesh

## Abstract

With a lack of population-specific reference intervals (RIs) in Bangladesh, this study aimed to determine RIs for complete blood count (CBC) and specific micronutrients, vitamin D (VitD) and zinc, and to assess the possible impact of prior history of SARS-CoV-2 infection on these parameters. Healthy participants (*n* = 1724) of both sexes aged ≥ 10 years from slum and non-slum areas of Dhaka city were sampled systematically. A total of 26 CBC parameters were determined using an automated hematology analyzer, whereas the micronutrients VitD and zinc were measured using automated immunoassay analyzers and atomic absorption spectrophotometry (AAS), respectively. Multiple regression analysis (MRA) was employed to determine the association of variations in reference values (RVs) of hematological and micronutrient parameters with sex, age, and slum residency of the participants. Practical significance of each factor was judged from partial correlation coefficients (r_p_) by setting its minimum effect size at |r_p_| = 0.2. The need to partition RVs by sex and age was assessed using the ANOVA-based standard deviation ratio (SDR). RIs were determined by parametric method after Gaussian transformation using the two-parameter Box–Cox formula, with or without the latent abnormal values exclusion (LAVE) method. By MRA, sex and age were significant source of variations for various CBC parameters, whereas slum residence was associated with increased levels of VitD and zinc Using the SDR ≥ 0.35 threshold, RVs were partitioned by sex to derive RIs for hemoglobin, hematocrit, red cell count and indices (MCH, MCHC), and plateletcrit, as well as for VitD and zinc. Comparing these RIs with those from the global collaborative studies indicated significant differences in erythrocyte and leukocyte parameters. These are the first population-specific RIs for urban Dhaka city, underscoring the need for country-specific RIs for accurate clinical use.

## 1. Introduction

Bangladesh grapples with regional health disparities influenced by factors like dietary habits, environmental conditions, and genetics [1]. Recognizing these variations is crucial for establishing unbiased reference intervals (RIs) to assess and monitor health across diverse regions and populations. RIs serve as essential tools in laboratory science, assisting health professionals in interpreting clinical results and making informed decisions for patient care. RIs for complete blood count (CBC) are indispensable for diagnosing and monitoring health conditions, including infectious diseases such as malaria and dengue, which may exhibit regional variations [2,3]. Furthermore, RIs for micronutrients like vitamin D (VitD) and zinc are essential, particularly in identifying (nutritional) deficiencies prevalent in specific regions or population groups. This data is vital for public health planning, aiding policymakers in making informed decisions about healthcare priorities and resource allocation [4,5,6,7]. Additionally, standardized RIs may enable participation in international health research, fostering collaboration and benchmarking against global standards [8,9,10]. However, there are currently no published RIs for hematological and biochemical parameters specific to the Bangladeshi population. Clinical laboratories in Bangladesh often rely on reference values (RVs) from reagent manufacturers and the medical literature, potentially overlooking regional variations in disease prevalence and treatment outcomes. This reliance on RVs largely from Europe and America, where reagents are sourced, may not accurately represent the unique characteristics of the Bangladeshi population in terms of age, sex, lifestyle, diet, genetics, and geographical location. Variation in parameters across regions highlights the importance of establishing context-specific RIs to prevent inaccuracies in clinical diagnoses and treatments based on RVs that may not apply to the local population.

RIs for CBC, VitD, and zinc are necessary for Bangladesh. Determining RIs requires precision, with steps such as identifying reference individuals and standardizing sample handling [11]. Despite efforts to include only healthy subjects, subclinical diseases of high prevalence persist in apparently healthy individuals, distorting the distributions of RVs [12]. Heterogeneity among reference individuals, lack of a perfect standard for normality, and variations in statistical methodology pose substantial challenges. The statistical method called latent abnormal values exclusion (LAVE) addresses the potential inclusion of individuals with subclinical conditions, such as latent anemia, in RI studies by excluding individuals with abnormal test results, thereby enhancing the accuracy and reliability of the determined RIs [13].

The primary objective of this study was to establish RIs for hematological parameters and selected micronutrients (VitD and zinc) in the urban Bangladeshi population and to compare these intervals with those reported internationally to identify population-specific characteristics. Additionally, our study aims to explore variations in hematological parameters and key micronutrient RIs, such as VitD and zinc, across age, sex, body mass index (BMI), and slum residency.

The exploratory goal of this research is to examine whether prior infection with the SARS-CoV-2 virus, the causative agent of the COVID-19 pandemic that emerged in 2019, is associated with significant changes in hematological parameters as well as micronutrient levels in previously infected individuals [14,15,16,17,18,19,20,21]. This inquiry holds particular implications, given that a substantial portion of our study population tested positive for SARS-CoV-2 in a cross-sectional epidemiological study that was conducted in Bangladesh between October 2020 and February 2021 during the pandemic [22]. With these objectives, we included apparently healthy individuals recruited in the survey. Based on a structured health-status questionnaire, we carefully selected only those individuals who were suitable for establishing the RIs.

## 2. Materials and Methods

### 2.1. Study Settings

We employed a combination of purposive and systematic sampling techniques to enroll participants under a SARS-CoV-2 serosurveillance study from October 2020 to February 2021. We deliberately selected large slum and adjacent non-slum areas in Dhaka to analyze the possible impact of household income status on establishing RIs in Bangladesh. This stratification serves as a proxy for extreme socioeconomic and structural variations; slum areas represent high-density, low-income settlements with shared utilities and severe overcrowding, which significantly alter pathogen exposure and baseline health metrics. Conversely, the neighboring non-slum areas are characterized by middle-to-high-income households residing in multi-storied buildings constructed with brick and concrete with independent household infrastructure. Selecting adjacent cohorts allowed us to control for overarching geographical and environmental variables while isolating the effects of socioeconomic disparities. We employed the Urban Health and Demographic Surveillance System (UHDSS) sampling frame for slum areas, and for non-slum areas, we initially conducted household listings and then randomly chose samples from them. We used a random-cluster sampling technique by organizing households into equal-sized clusters, which were then randomly selected.

We finalized a structured questionnaire, which was synchronized with a Tablet/Android-based electronic questionnaire linked to a server following each interview. The data collection process was managed using the SQLite program, and data cleaning and management involved the use of the SQLite browser, SQL, and Visual FoxPro. We collected comprehensive participant information, including data on age, sex, education, employment status, family income, chronic diseases, and any COVID-19-like symptoms experienced in the past month.

### 2.2. Participant Enrollment

**Figure 1:**
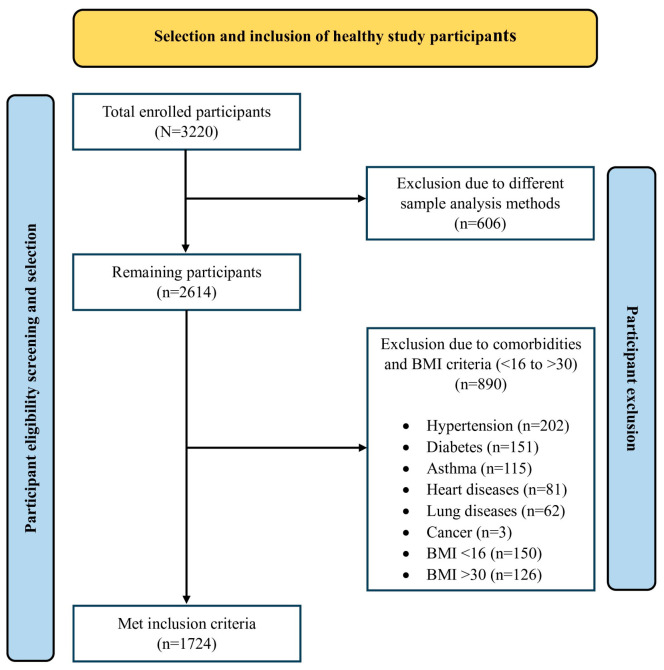
Flow diagram representing the inclusion/exclusion of the study participants.

Among the 2614 participants enrolled, we employed a rigorous selection process to identify healthy individuals. First, we excluded those with any comorbidities, as diagnosed by a physician, which included a history of stroke, heart disease, diabetes, lung diseases, asthma, hypertension, and cancer (*n* = 617). Furthermore, we excluded participants with a BMI less than 16 (*n* = 150) and those with a BMI greater than 30 (*n* = 123). Consequently, we determined the RIs based on a final cohort of 1724 participants (Figure 1).

Height and weight were measured by trained nurses or medical technologists during the visit using a portable stadiometer (Seca, Stratford, CT 06615, USA) and a digital scale (RFL, Dhaka, Bangladesh). Blood pressure was measured twice using a manual BP machine (ALPK 2, Tokyo, Japan) in a sitting position with a 15 min rest in between. The averages of the two measured values were utilized.

### 2.3. Specimen Collection and Processing

Blood samples (7.5 mL) were collected by a trained phlebotomist; 5.0 mL was placed into a trace element-free sodium heparin tube (Greiner Bio-One GmbH, Kremsmünster, Austria) to measure 25-hydroxyvitamin D and zinc, and 2.0 mL into hematology tubes with spray-coated K2EDTA (BD, Franklin Lakes, NJ 07417-1880, USA) to measure the concentration of hemoglobin (Hb). The samples were transported to the laboratory within 2–3 h. Upon receipt in the laboratory, Hb was measured from the hematology tube. Plasma was separated from the heparin tubes by centrifugation at 3500 rpm for 10 min using a refrigerated centrifuge, apportioned into 600 µL aliquots, and stored at −80 °C until use.

### 2.4. Specimen Analysis

#### 2.4.1. Assessment of Hematological Markers

CBC was measured on an automated 5-part (Diff. 26 parameter) hematology analyzer (XS-800i, Sysmex Corporation, Kobe, Japan) to determine 26 whole-blood diagnostic parameters. These include parameters related to erythrocyte: hemoglobin (Hb), red blood cell (RBC) count, hematocrit (Ht), red cell indices (MCV, MCH, MCHC), red cell distribution width (RDW-SD, RDW-CV); parameters related to absolute leucocyte count: total count (WBC), neutrophil (Neu), lymphocyte (Lym), monocyte (Mon), eosinophil (Eos), basophil (Bas); parameters related to differential leucocyte count: Neu%, Lym%, Mon%, Eos%, Bas%; parameters related to platelet: platelet count (PLT), mean platelet volume (MPV), platelet distribution width (PDW), platelet large cell ratio (P-LCR), and plateletcrit (PCT).

The analyzer was installed with an online calibration system for reliable internal quality control. E-check Tm (XE) level-2 (Sysmex) was used in each lot/day to verify accuracy and precision. Randomly selected samples were forwarded to the hematology laboratory at icddr,b for cross-checking and verification of CBC reports.

#### 2.4.2. Assessment of Micronutrient Parameters

VitD was measured by competitive chemiluminescent protein-binding assay on Roche automated immunoassay analyzers Cobas e601 using the Vitamin D Total kit (Roche Diagnostics, GmbH, 68305 Mannheim, Germany). This assay employed a vitamin D-binding protein (VDBP) as the capture protein, which binds 25-OH D3 and 25-OH D2 and is reported as total 25-hydroxyvitamin D. This method had been standardized against LC-MS/MS. Commercial control based on human serum at two concentration levels was run for each lot/day to monitor the assay’s accuracy and precision. To ensure the quality of VitD results, the laboratory participates in the VITAL-EQA Proficiency Testing Program organized by the CDC, which is performed twice a year.

Plasma zinc was measured by flame atomic absorption spectrophotometry (AAS; Shimadzu AA-6501S, Kyoto, Japan). Plasma was diluted with de-ionized water and aspirated directly into the flame of the AAS, along with a set of working standard solutions (Kanto Chemical Co., Inc., Tokyo, Japan). Readings are recorded at 213.9 nm using a zinc hollow-cathode lamp and an air–acetylene flame. Bi-level trace elements serum toxicology control (UTAK Laboratories Inc., California, CA 91355, USA) was run in each lot to check accuracy and precision. To ensure the quality of results, the laboratory participates in the College of American Pathologists (CAP) Proficiency Testing Program, which is performed three times a year.

The Elecsys^®^ Anti-SARS-CoV-2 assay was used on the Cobas-e601 immunoassay analyzer (Roche Diagnostics GmbH, Mannheim, Germany) to measure nucleocapsid (N) antigen-specific IgM and IgG antibodies in plasma, indicating recent or past SARS-CoV-2 infection. Participants were enrolled as apparently healthy individuals; however, their previous SARS-CoV-2 infection status was determined retrospectively based on antibody titers. Information on the duration since prior infection was available from participant records, and some individuals had experienced asymptomatic infection. Serological responses were classified based on the cutoff index (COI): reactive/seropositive (COI ≥ 1.0) and non-reactive/seronegative (COI < 1.0) [22].

### 2.5. Ethical Approval

The study was approved by the institutional review board (PR-20070, dated 1 September 2020) of the icddr,b. Written informed consent was obtained from adult participants, while assent was obtained from 10–17-year-old children and consent was obtained from their parents.

### 2.6. Statistical Analyses

#### 2.6.1. Sources of Variation Analysis

To explore sources of variation in each analyte, we performed multiple regression analysis (MRA) [23]. RVs of each analyte were set as an objective variable. The following factors were included constantly in the regression model as explanatory variables: sex, age, BMI, post-SARS-CoV-2 infection status (post-CoV: binary 1 = infection (+), 0 = (−)), and residence in slum areas (binary: 1 = slum, 0 = non-slum). Smoking and alcohol consumption of participants were not evaluated as potential sources of variability, which is a limitation of this study. The magnitude of association of each factor with the objective variables was expressed as the standardized partial regression coefficient (stdβ), which corresponds to the partial correlation coefficient (r_p_) that takes a value between −1.0 and 1.0. In reference to Cohen’s criteria on the effect size of the correlation coefficient, we regard the association as weak but significant when 0.2 ≤ |r_p_| < 0.3, moderate when 0.3 ≤ |r_p_| < 0.5, and strong when 0.5 ≤ |r_p_| [24].

#### 2.6.2. Partitioning Criteria for Reference Values (RVs)

To assess the necessity of stratifying RVs by sex and age, we calculated the standard deviation ratio (SDR), which represents the ratio of the variation between subgroups’ standard deviations (representing the deviation of subgroup means from the overall mean) to the standard deviation between individuals (approximately one-fourth of the width of the RI). To determine whether sex and age subgroup differences warranted partitioning, we conducted a two-level nested analysis of variance (ANOVA). In this analysis, we computed the standard deviation for sex (SDRsex) and age group (SDRage) after categorizing age into the following groups: 10–18 years, 19–39 years, 40–59 years, and those above 60 years.

We also calculated the SDRage separately for each sex using a one-way ANOVA since age variation differs between sexes. We used an SDR value of 0.35 as a threshold to guide our decision on whether to partition RVs by sex or age [13].

#### 2.6.3. Derivation of RIs

To facilitate a comparative analysis, RIs were calculated using both parametric and non-parametric approaches before and after application of the LAVE method, as detailed in Ichihara et al. [13,25]. To summarize, the non-parametric method entailed arranging the data in ascending order and identifying the RI lower and upper limits (LL and UL) as the values corresponding to the 2.5th and 97.5th percentiles, respectively. In contrast, the parametric method involved transforming the RVs to a Gaussian distribution using the two-parameter Box–Cox power transformation formula [25,26]. Using the mean and standard deviation in the transformed scale (M_T_, SD_T_), the central 95% limits can be calculated as M_T_ ± 1.96 SD_T,_ if the Gaussian transformation is successful. The reverse transformation of the limits gives the central 95% limits (or LL and UL) in the original scale.

The LAVE method is an iterative optimization procedure applied when mutually related analytes are tested simultaneously [13,25]. Initially, RIs are determined independently for each analyte. From the second computation, any individual who had two or more results outside the RIs derived in the previous computation among the reference tests was excluded. This process was repeated six times, at which point the RIs were nearly stable. Of note, the need for exclusion of an RV is determined using a set of pre-defined reference tests, but not the test whose RI is being determined.

In this study, we used the following 9 analytes as reference tests for use in the LAVE procedure—Hb, MCV, RDW-CV, WBC, Lym%, Mono%, PLT, and PDW—which were chosen as having slight-to-moderate correlations among them: i.e., analytes with strong mutual correlations were excluded to avoid trimming too many peripheral values.

#### 2.6.4. Bias Assessment at Reference Limits

We calculated the bias ratio (BR) of the lower limit (LL) and upper limit (UL) of the RI before and after implementing the LAVE procedure by using the following formulas:

$${BR}_{LL}=\frac{{LL}_{+}-{LL}_{-}}{({UL}_{+}-{LL}_{+})/3.92}\;\;{BR}_{UL}=\frac{{UL}_{+}-{UL}_{-}}{({UL}_{+}-{LL}_{+})/3.92}$$

Here, ${LL}_{+}$ and ${UL}_{+}$ represent the RI limits after applying LAVE, while ${LL}_{-}$ and ${UL}_{-}$ denote the RI limits without LAVE [27]. We designated 0.375 as the threshold for BR, analogous to the allowable limit of analytical bias in clinical chemistry, to define a significant change in either LL or UL following the implementation of LAVE [28].

We also calculated BR in evaluating between-sex differences in LL and UL in case SDRsex is within the threshold of 0.35, but between-sex differences at LL or UL are apparent by using the following formulae:

$${BR}_{LL}=\frac{{LL}_{M}-{LL}_{F}}{({UL}_{MF}-{LL}_{MF})/3.92}\;\;{BR}_{UL}=\frac{{UL}_{M}-{UL}_{F}}{({UL}_{MF}-{LL}_{MF})/3.92}$$

where UL_M_, UL_F_, and UL_MF_ represent the ULs for males, females, and the combined group, respectively, and LL_M_, LL_F_, and LL_MF_ denote the corresponding LL. In using BR for evaluating between-sex bias, we adopted 0.50 (≈0.35 × $\sqrt{2}$) because SD of two values (x_1_ and x_2_) is 1/$\sqrt{2}$ of the bias of the two values (|x_1_ − x_2_|) [29].

## 3. Results

### 3.1. Participants’ Demographic Characteristics

The average age of the slum participants was 30.5 years, while in the non-slum area, the average age was 29.0 years. More than half of the participants (53.4%) were aged 18–40 years, followed by adolescents aged 10–17 years (24.2%). The proportions of females in slum and non-slum areas are 54.7% and 58.2%, respectively. Additionally, the percentage of participants with a reactive antibody response was higher in the slum area (74.2%) compared to the non-slum area (64.8%) (Table 1).

In the slum area, 26.9% participants were overweight, while 17.7% were underweight. On the other hand, 38.1% participants were overweight, and 10.1% were underweight in the non-slum area. Regarding occupation, students and homemakers were more common in non-slum areas, while self-employed and unemployed individuals were more prevalent in slum areas. Family income differed markedly between the two residences, with over half (56.6%) of slum residents reporting a monthly income below 20,000 BDT, whereas nearly half (49.7%) of non-slum participants reported an income between 40,000 and 70,000 BDT (Table 1).

### 3.2. Sources of Variation Analysis by MRA

Sources of variation (SVs) for each analyte were assessed using a minimal threshold of |rp| ≥ 0.15. Of these, age, slum residency (binary), and BMI were identified as the most consistently significant factors across analytes. Age-related variations were prominent in both sexes. Among males, an age-related decrease in RVs was observed for RBC (r_p_ = −0.256), Lym% (−0.209), and PCT (−0.166). In contrast, age-related increases in RVs among males were observed for VitD (0.344), MCV (0.268), MCH (0.258), RDW-SD (0.246), and Neu% (0.194). Among females, significant negative age-related effects were evident in RBC (−0.240), Hb (−0.151), Ht (−0.174), and WBC (−0.194), while VitD (0.203) and RDW-SD (0.138, near threshold) showed positive associations. However, no significant effects of SARS-CoV-2 on the CBC and micronutrient parameters were observed (Table 2).

Slum residency was positively associated with higher VitD levels (male: 0.384; female: 0.326) and higher zinc levels (male: 0.222; female: 0.255). On the other hand, in females, slum residency was associated with lower platelet parameters, particularly MPV (−0.192), P-LCR (−0.208), and PDW (−0.233), whereas no such associations were observed in males (Table 2).

BMI-related increase was observed in males for RBC (r_p_ = 0.162) and PDW (0.158), and in females for WBC (0.178). Finally, reactive SARS-CoV-2 antibody status had essentially exhibited no impact on hematological parameters, although a slight association with zinc was observed in males (r = 0.162) (Table 2).

### 3.3. Sex- and Age-Related Changes in Reference Values (RVs)

**Figure 2:**
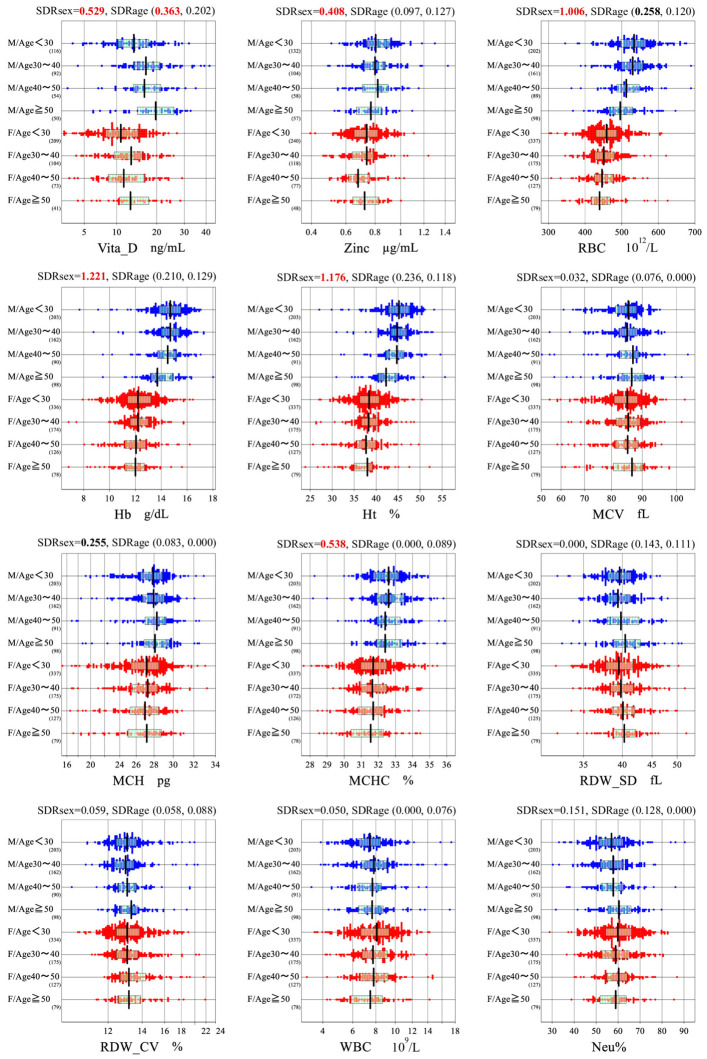

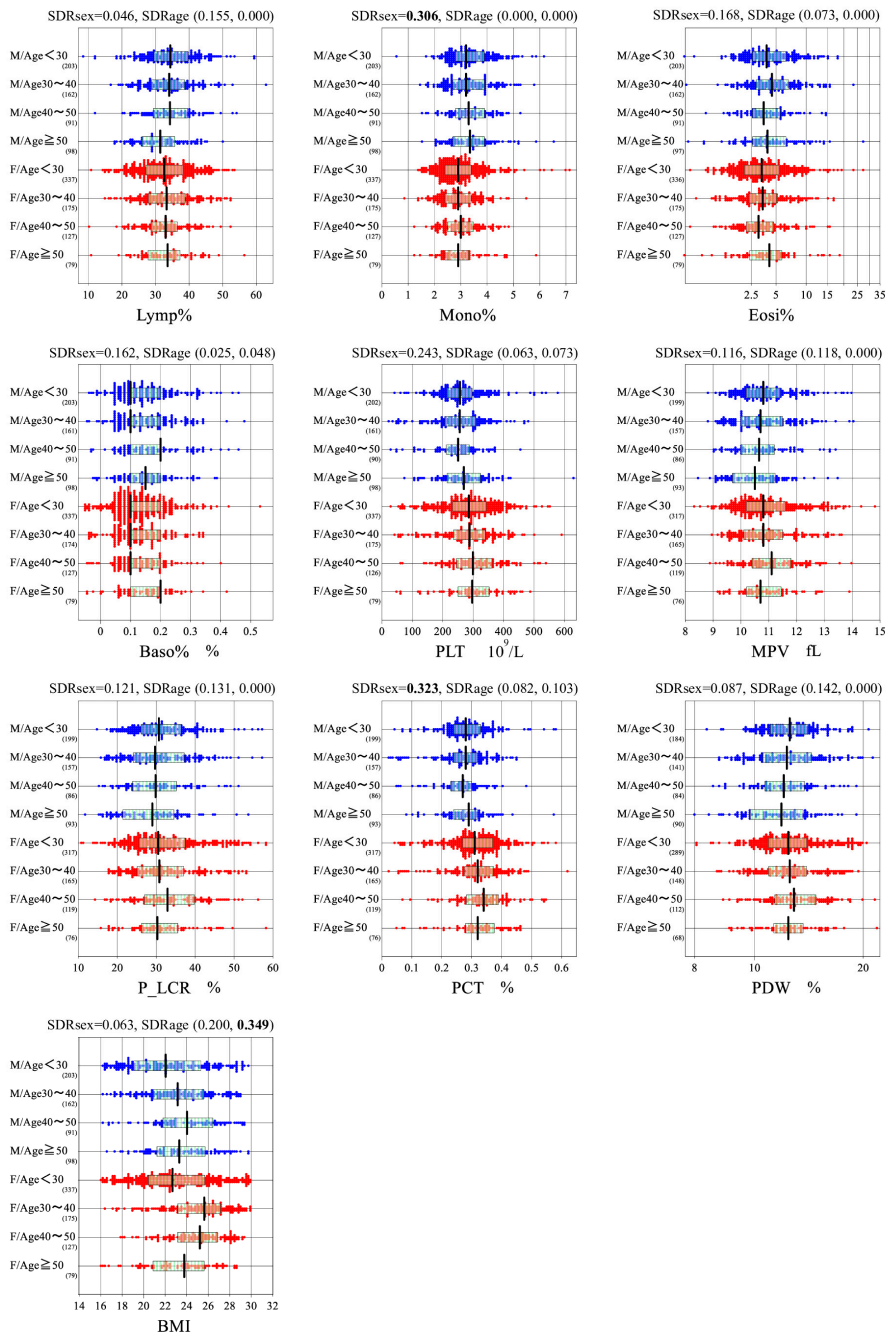
Age- and sex-related changes in RIs for CBC and micronutrient (VitD and zinc) parameters in the study population. The box in the center of each scattergram indicates the mid-50% range of RVs, and its central vertical bar represents the median.

Table 3 illustrates the extent of sex disparities, as indicated by SDRsex, derived from a two-level nested ANOVA with age as a covariate. Considering SDR ≥ 0.35 as the partitioning threshold, between-sex differences were conspicuously observed in VitD (SDRsex = 0.529), zinc (0.402), RBC (1.006), Hb (1.221), Ht (1.176), and MCHC (0.538), while minor differences were noted for MCH (0.255) and Mon% (0.306). As for the between-age subgroup differences, there was no analyte exceeding the threshold of 0.35, but an appreciable age-related increase was noted in RVs of VitD (male: 0.344; female: 0.203) and slightly in RVs of Hb (female: −0.151) and Ht (female: −0.174). For RBC and MCHC, only sex-related changes were significant; for MCH, only age-related changes were significant in males. It is of note that, although we did not observe between-sex differences in RVs for RDW-SD and RDW-CV, there was a conspicuous tendency for a wider spread of their RVs in females compared to males. This finding indicates that SDRsex represents a between-subgroup difference at the central part of the RVs but does not reflect the difference in the spread of the RVs (Figure 2).

It is of note that the marginal significance of sex-related change was observed for zinc. For the remaining parameters, neither age nor sex emerged as a significant source of variation, leading us to decide against partitioning their RVs (Table 3).

### 3.4. Derivation of RIs for the Hematological and Micronutrient Parameters

**Figure 3:**
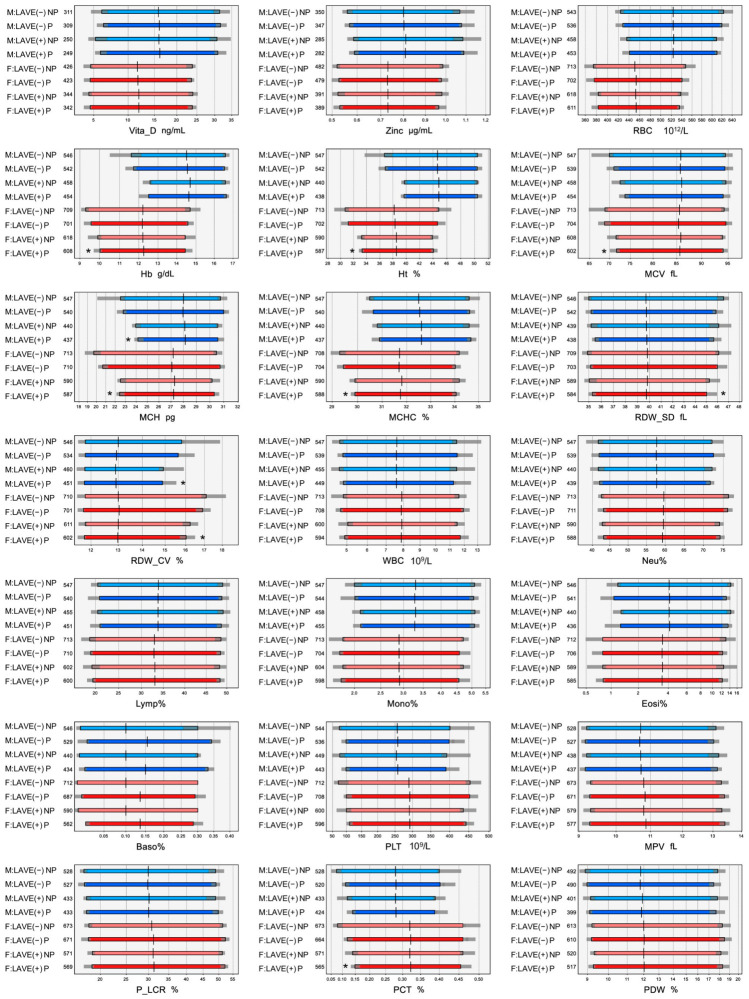
Comparison of CBC and micronutrient RIs derived from four methods, i.e., P and NP, with or without the LAVE method. The gray shading at RI limits represents 90% confidence intervals. The mark * indicates significant differences in RI limits (|BR| ≥ 0.375) caused by the LAVE procedure.

RIs were computed using four approaches: parametric (P) and non-parametric (NP) methods, with or without the LAVE method. Figure 3 presents the comparisons of RIs by the four methods for all analytes. Overall, RIs by P and NP methods are comparable in most analytes. However, compared to the P method, the NP method exhibited downward LL shift in VitD, MCV, and PCT; upward UL shift in RDW-CV, WBC, and Bas%; broadened RI for Zn, RDW-SD, and PLT (Supplementary Table S1 and S2). In addition, 90% CI of RI limits was broader in VitD, Zn, RDW-CV, WBC, Eos%, PLT, and PCT. Consequently, we adopted the P method in computing RIs for all analytes.

The effect of the LAVE method in narrowing the RI width by the P method was appreciably (|BR| > 0.375) observed for Hb, Ht, MCV, MCH, MCHC, RDW-CV, and PCT as indicated by * in the figure. Additionally, minor but noticeable degrees of the LAVE effect (0.10 ≤ |BR| < 0.375) were observed for zinc, WBC, Eos%, Bas%, PLT, etc. (Figure 3).

Table 4 lists the RIs adopted for all analytes. In determining RIs with or without stratification by sex, our primary criteria involved SDRsex. However, we also accounted for the bias ratio (BR) at the RI limits (BR_LL_ and BR_UL_), as presented in Supplementary Table S1. For partitioning RVs by sex, guided by SDRsex ≥ 0.35 as the threshold, the selected analytes were VitD, RBC, Hb, Ht, and MCHC. The parametric method with/without the LAVE method provided refined RIs for micronutrients and CBC parameters in the Bangladeshi population (Table 4, Supplementary Table S1).

For VitD, the RIs were markedly low across all groups, with females showing the lower-side shift in RIs (4.02–24.2 ng/mL) compared to males (4.82–30.7 ng/mL). Zinc levels were comparable between sexes, though males showed slightly higher upper limits (1.10 µg/mL vs. 1.00 µg/mL), as indicated by the marginal SDRsex of 0.308.

For hematological parameters, RIs for RBC, Hb, and Ht were significantly lower in females, with Hb ranging from 10.2 to 14.4 g/dL in females compared to 12.1–16.7 g/dL in males. Similarly, Ht levels were lower in females (33.6–44.1%) than in males (38.4–50.6%). The width of MCHC RI in females was narrower than that of males. RDW-CV RIs indicated greater variability in females (11.7–15.4%) than in males (11.7–14.7%). WBC RIs were higher in females (4.92–12.2 × 10^9^/L) compared to males (4.74–11.2 × 10^9^/L), primarily due to higher Neu% (42.4–74.8% in females vs. 38.8–72.4% in males). Conversely, Mon% was slightly higher in males. Platelet indices also demonstrated sex-specific differences, with females showing higher PLT (113.1–450.6 × 10^9^/L vs. 97.2–412.0 × 10^9^/L in males). MPV, P-LCR, and PCT also showed slightly elevated RIs in females (Table 4).

### 3.5. Differences Between Global RIs and Bangladeshi Population-Specific Ranges

**Figure 4:**
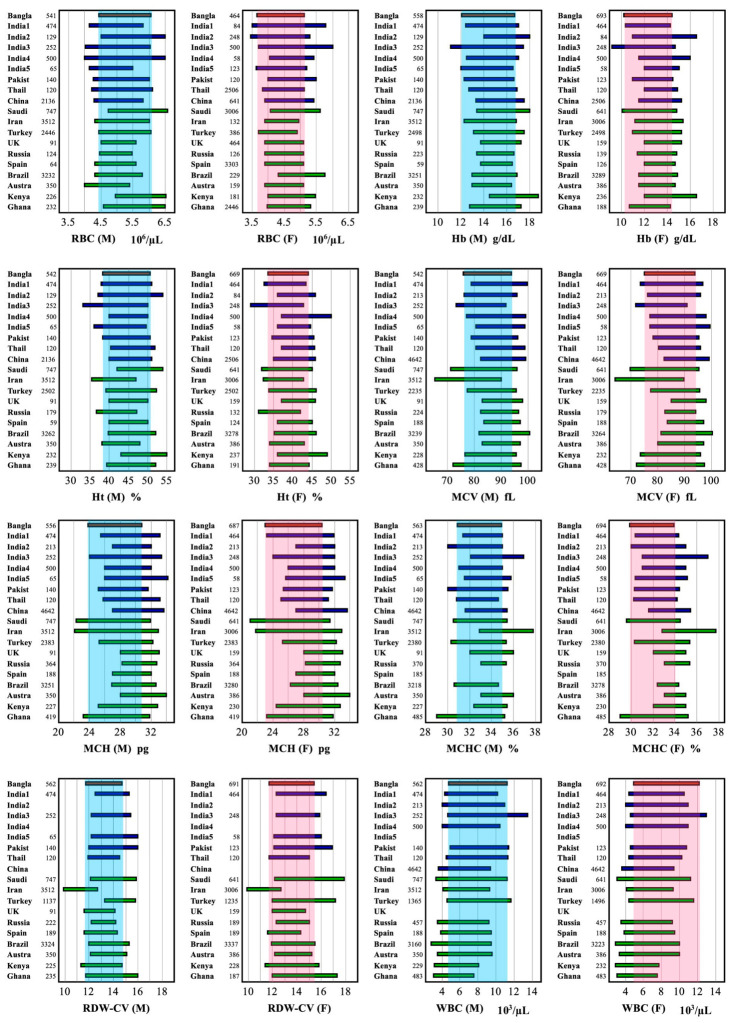

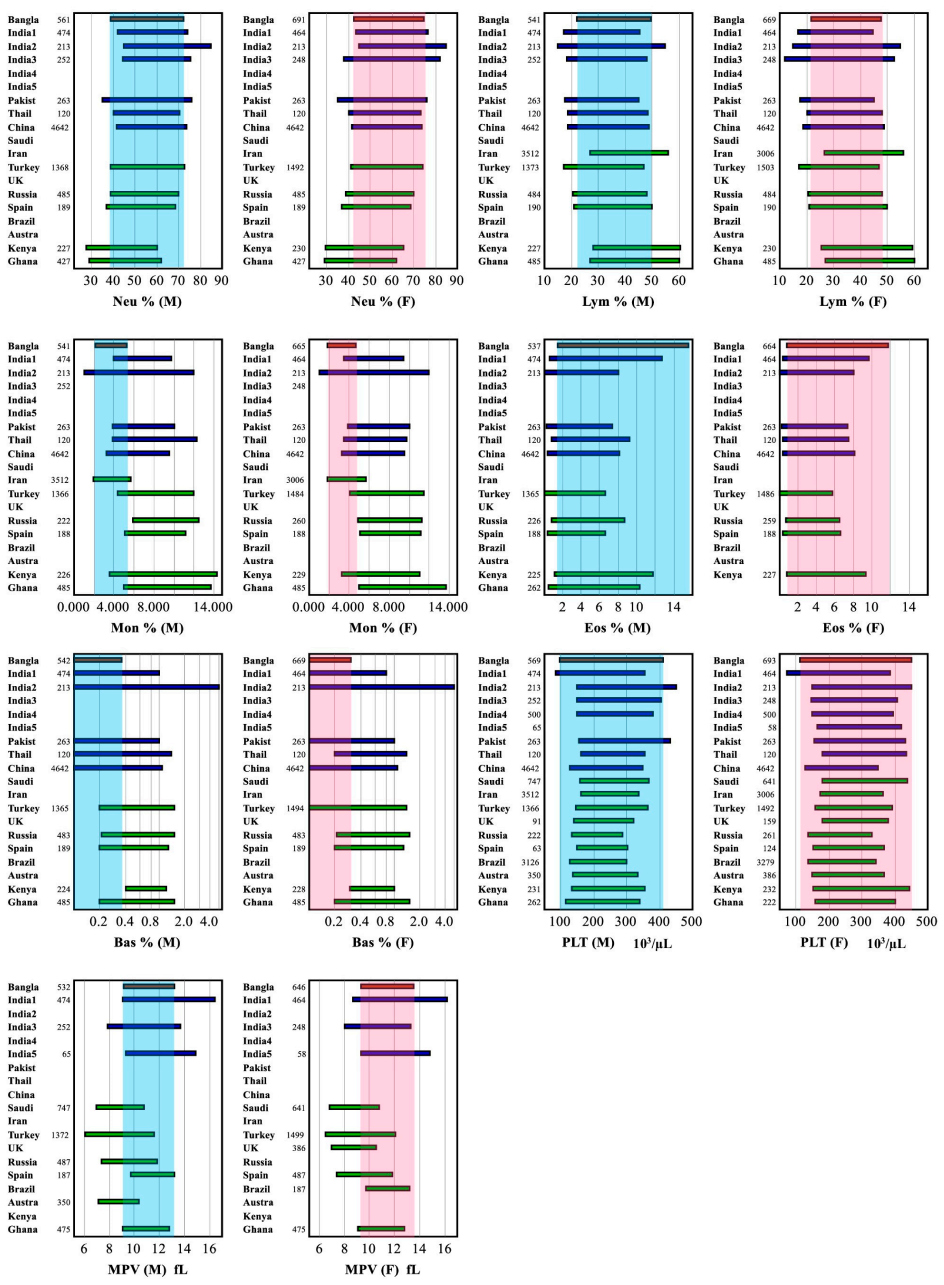
Comparison of RIs for CBC parameters in male and female participants: present study vs. other studies conducted worldwide. The blue and red back-shades represent Bangladeshi RIs established in this study.

The mean Hb concentration in the healthy Bangladeshi cohort was 12.8 g/dL, with a lower reference limit of 10.2 g/dL. This lower limit is notably below the thresholds reported in Western populations (e.g., USA: 12.8 g/dL) and comparable to the range observed in India (10.5–14.8 g/dL), suggesting a population-level tendency toward lower baseline Hb. The Mean Corpuscular Volume (MCV) was 81.54 fL, within the range of 76.24–95.21 fL, and closely matching the typical values of other Asian populations (e.g., China: 82.7–93.5 fL) (Figure 4) (Supplementary Table S3).

The RIs for hematological parameters in the Bangladeshi population exhibit a distinct profile compared with global data, characterized by significantly lower upper limits for monocytes (4.62–5.27%) and basophils (0.31–0.36%) than those observed in India (9.7% and 1.01%) and Kenya (14.3% and 1.2%). Conversely, the upper limit for eosinophil is notably elevated at 11.8–15.6%, nearly doubling the thresholds found in Spain (6.6%) and Thailand (9.2%) (Table 4) (Supplementary Table S3).

For zinc, the established RI (0.55–1.05 µg/mL) had a slightly lower upper bound than the Ethiopian and US ranges (Figure 4). The most significant finding was related to VitD, with an RI of 4.03–27.5 mg/mL. The calculated lower reference limit for VitD was 4.03 ng/mL, which is dramatically lower than the typical insufficiency cutoffs of 20–30 ng/mL used internationally (Supplementary Table S3).

## 4. Discussion

### 4.1. Key Methodological Considerations Specific to This Study

Our study, involving a large and diverse population from slum and non-slum areas of Dhaka, took a step towards establishing RIs for CBC parameters and key micronutrients (VitD and zinc).

The demographic characteristics of our study participants revealed significant variations in several analytes based on age and sex. For example, VitD, MCV, MCH, RDW-SD, and zinc displayed age-related changes, particularly among males. Sex-related differences (SDRsex ≥ 0.35) were observed in RBC, Hb, Ht, and MCHC. In addition, despite a subthreshold level of SDRsex, a large between-sex BR of LL or UL exceeding the threshold of 0.50 (≈0.35 × $\sqrt{2}$) was observed in RVs for PLT, Mon%, Eos%, and Bas% (Figure 2) (Table 2 and Table 3). These variations underscore the importance of considering demographic factors when interpreting laboratory results.

### 4.2. Comparison of the Current Study Findings with Existing Studies: Concordance and Explanatory Factors

In examining hematologic parameters associated with erythrocytes, such as Hb, RBC, Ht, MCV, MCH, and MCHC, males typically exhibited higher LL and UL values than females, which can be attributed to various physiological factors and the influence of sex hormones (Table 4) [29,30,31]. Additionally, factors such as higher muscle mass and androgen-mediated stimulation of erythropoiesis in males may contribute to these differences.

Due to limited publication on CBC variations and RIs in the Bangladeshi population, comparisons were restricted. However, the RIs proposed by Bhowmik et al. in a cross-sectional study in Dhaka show some comparability with our findings. Notably, the LLs for both males and females were lower in their study, possibly due to limited geographic variations, a narrower age range (18–45 years), and the use of only simple parametric methods [32]. Compared to studies from the other South Asian region, our study findings showed similarities in LL and UL hematological RI limits in some parameters and dissimilarities in other parameters. For instance, the study by Rahar et al. in a seemingly healthy population in Delhi, India, reported Hb, RBC, Ht, and MCHC levels similar to our findings; however, the RIs for other parameters differed considerably from those observed in our study [33]. A study conducted in Kolkata, India, also reported significant variations in other erythrocyte parameters, including Ht, MCHC, MCV, MCH, and RDW [34]. In a Pakistani population, Hb and RBC RIs were similar to our findings, whereas Ht, MCHC, MCV, MCH, and RDW differed substantially, highlighting the need for country-specific RIs (Figure 4) (Supplementary Table S3) [35]. When we compared the findings with other Asian countries, similar lower and upper limits for Hb and RBC were reported in studies from Thailand and Iran; Hb RIs for females in studies from Saudi Arabia and China were also close to those observed in our study [36,37,38,39]. Our RDW-CV RIs are consistent with findings from studies in Thailand and Korea; however, other Asian studies report notable differences in RIs for several hematological parameters [36,37,38,40,41]. Moreover, RIs for erythrocyte parameters from studies in Europe, America, and Africa often differ substantially from ours, though RBC, MCHC, MCH, and RDW show closer agreement in some non-Asian studies (Figure 4) (Supplementary Table S3) [42,43,44,45,46,47].

Similarities were mostly found in populations that share similar physiological and dietary characteristics (Figure 4). The variations in RI limits with other studies were mainly due to variations in sample size, participant age, eligibility criteria, and statistical methods [35,48,49,50,51]. Our study excluded participants with comorbidities or BMI <16 or >30 (Figure 1). In contrast, other studies implemented different criteria, for example, a BMI greater than 35 kg/m^2^, the use of medications for chronic diseases, being a known carrier of HBV, HCV, or HIV, daily ethanol consumption, smoking, etc. [46]. Tobacco smoking and alcohol consumption are associated with increased erythrocyte-related parameters [52,53,54,55,56,57,58]. However, smoking and alcohol consumption data were not collected in this study, which constrains the comparability of our findings with those of other studies.

The application of the LAVE technique, which iteratively excludes abnormal test values linked to subclinical conditions, further enhances the validity of our proposed RIs by minimizing the risk of variations caused by underlying clinical conditions that went undetected during participant screening [59]. The impact of the LAVE procedure on lowering the upper limit of the RI was clear, providing a more appropriate representation of the healthy population, as supported by various published studies conducted in different populations worldwide [46,47,60].

Several erythrocyte RIs proposed in our study align with those from LAVE-based studies elsewhere; for example, RBC RIs match findings from Saudi Arabia, Kenya, Turkey, and Japan, while other parameters differ significantly [46,60,61]. Notably, the RIs of Ht for females by Borai, Bawua, and Ichihara et al. closely matched our findings [47,60]. Additionally, our RIs for RDW are consistent with those reported by Omuse et al. in their study of the Kenyan population [46]. The RIs for other erythrocyte parameters, however, vary significantly from those proposed in our study (Supplementary Table S3) [47,60]. This discrepancy may arise from variations in factors influencing RIs, such as ethnic diversity among study participants and the geographic characteristics of the study areas.

Erythrocyte RIs are strongly affected by ethnic differences and genetic variations. For example, Black populations, especially African Americans, typically have lower mean Hb and Ht with smaller RBCs, leading to lower MCV compared to white populations [62]. Furthermore, the disparities observed between the RIs in our study and those of similar studies may also be attributed to dietary habits and environmental factors. In Bangladesh, especially among slum populations, nutrient-poor diets increase malnutrition risk, leading to lower Hb, Ht, RBC, MCH, and MCV, reflecting impaired erythropoiesis and anemia-like effects [63,64,65]. Latent iron deficiency (LID), defined by depleted iron stores in the absence of anemia, produces subtle alterations in erythrocyte indices including MCV, MCH, and RDW prior to a decline in Hb [66]. The inclusion of individuals with undetected LID in our study may shift RIs toward iron-depleted values, thereby masking early erythropoietic abnormalities and reducing the diagnostic sensitivity of routine CBC parameters [67].

Environmental pollution, particularly air pollution in Dhaka city, may also contribute to hematological variation among our study participants, with slum residents facing the greatest exposure risk. Evidence from multiple studies consistently links air pollution to adverse alterations in RBC counts and reductions in both RBC and Hb levels, primarily through oxidative stress and inflammation driven by reactive oxygen species and pro-inflammatory cytokines [68,69,70].

Regarding WBC-related hematological parameters, our study suggested higher RIs for both the lower and upper limits of the WBC and neutrophil counts in females compared with males. However, for other WBC parameters, including lymphocytes, monocytes, eosinophils, and basophils, the values were higher among male participants (Table 4). These sex-related variations in WBC parameters contradict the findings of several similar studies, likely because leukocyte composition is more influenced by age than by sex. A variety of factors, particularly sex hormones such as estrogen and androgen, play a significant role in shaping immune responses and leukocyte composition [71,72]. For WBC counts, the RIs established in our study for both males and females are notably higher than those reported in studies conducted across Asia, Europe, Africa, Australia, and North America (Figure 4) [39,42,46,47,61,73,74,75,76,77]. Additionally, significantly elevated RIs for lymphocytes, neutrophils, eosinophils, and monocytes were observed compared with similar studies from various continents (Supplementary Table S3). While conclusive data supporting the notion that the people of the Indian subcontinent, particularly the Bangladeshi population, tend to exhibit higher leukocyte counts in general is lacking, a study involving blood donors found that Indians and Black individuals exhibit higher absolute lymphocyte counts than their white counterparts [78].

The RIs established by this study indicate significantly lower upper limits for basophils and monocytes compared to the global data (Table 4) (Supplementary Table S3). These discrepancies may be attributed to several factors, including ethnic and genetic variations that result in hematological differences from Caucasian and African populations, as well as inherited traits that affect white blood cell distribution [79]. Nutritional factors also play a critical role, as diets in Bangladesh often contain lower levels of certain micronutrients, such as zinc and VitD, which are known to suppress the production of monocytes and basophils, thereby contributing to the reduced RI values within this demographic [80].

The upper limit for eosinophil (11.8–15.6%) is nearly double the thresholds found in Spain (6.6%) and Thailand (9.2%), which may reflect unique regional environmental and health influences (Table 4) (Supplementary Table S3). The higher RI values for eosinophils may be attributed to chronic exposure to helminthic parasites commonly found in tropical regions like Bangladesh, where sanitation and water quality challenges are prevalent [81,82]. Such exposures likely stimulate eosinophil elevation as an adaptive immune response. Environmental pollution, including air pollution and environmental allergens, e.g., dust, which are major problems in urban Bangladesh, especially in Dhaka, may also impact eosinophil counts [83,84,85].

In this study, we observed a lower LL for PLT, whereas the UL was higher than in various Asian and non-Asian studies (Figure 4) (Supplementary Table S3). This discrepancy may stem from several factors, including the use of the LAVE method to mitigate the effects of subclinical anemia and macrothrombocytopenia, a genetic condition often underdiagnosed in certain Indian regions, and endemic diseases such as dengue, malaria, and enteric fever, which can reduce PLT [86,87].

The current study sought to establish RIs for two essential micronutrients, zinc and VitD, in the studied population (Table 4). The proposed zinc RIs closely align with those reported by Barman et al. (overall: 0.6–1.2 µg/mL; male: 0.59–1.25 µg/mL; female: 0.5–1.03 µg/mL), in Bangladeshi adults aged 18 to 65 years [88]. However, the lack of published zinc RIs from other Asian countries with similar genetics, diets, and lifestyles limits broader comparisons.

For serum 25(OH)D, the proposed RIs in males (4.82–30.7 ng/mL) and females (4.02–24.2 ng/mL) are lower than international standards, reflecting widespread VitD deficiency in Bangladesh (Table 4). These lower ranges reflect severe deficiency, with the upper limit for males aligning with insufficiency [89]. The scarcity of studies establishing reference ranges for VitD in the Bangladeshi population, as well as in neighboring countries with similar genetics, geography, lifestyle, and dietary habits, limited the comparative analyses of our proposed intervals.

Several factors may act as potential confounders affecting VitD levels. Despite being a tropical country, sunlight exposure may be reduced due to indoor lifestyles among city dwellers, cultural or religious concealing clothing, and darker skin pigmentation, all of which limit VitD synthesis [90,91,92,93]. Inadequate dietary intake of VitD is another significant contributor to low levels of VitD, particularly among low-income individuals, as South Asian countries like Bangladesh have lower consumption of fortified foods and VitD supplementation compared with North American and European countries [94,95]. Age and sex also influence VitD levels, as indicated by the RIs proposed in this study. Women exhibit lower VitD levels due to indoor lifestyles, cultural dress norms, sunscreen use, and dietary habits, while VitD deficiency is more prevalent among older people due to decreased synthesis of dehydrocholesterol, impaired kidney and liver function, and reduced sunlight exposure from homebound behaviors [96,97,98].

The study found no significant effect of recent or prior SARS-CoV-2 infection, as determined by anti-N antibody titers, on RIs of CBC parameters, VitD, and zinc (Table 2). However, the cross-sectional design cannot fully capture post-infectious trajectories, timing since exposure, or low-grade inflammatory states that might subtly affect hematologic or micronutrient measures over time. Moreover, the study timeline (October 2020 to February 2021) strictly predates the introduction and rollout of the national SARS-CoV-2 vaccination program in Bangladesh, indicating no confounding risk from vaccine-induced seroreactivity or vaccine-driven changes to hematologic and micronutrient parameters in this data [22].

### 4.3. Strengths and Limitations of This Study

This study has several strengths. A key component of our research is the utilization of multiple regression analysis (MRA) following the guide provided by C-RID, which revealed that age and locality (slum vs. non-slum) are significant sources of variation for various analytes (Table 2) [25]. Additionally, the use of the LAVE method to exclude alterations in RVs related to subclinical diseases enhanced the accuracy and reliability of the study’s findings. The study recruited participants exclusively from slum and adjacent non-slum areas of Dhaka city, capital of Bangladesh, intentionally excluding rural regions that differ in environmental conditions, diet, and lifestyle from urban settings.

Our study has a number of limitations as well. First, we analyzed SARS-CoV-2 seropositive status as a binary variable rather than using antibody titers. Therefore, we could not evaluate the influence of antibody titers, time since infection, and severity of symptoms on hematological and micronutrient parameters. Moreover, participants with symptoms were excluded from this analysis. However, since our study period (October 2020 to February 2021) predates the rollout of the national COVID-19 vaccination campaign in Bangladesh, there were no effects of vaccination on SARS-CoV-2 seropositivity status; anti-SARS-CoV-2 seropositivity reflects natural infection only. Secondly, we did not collect information about smoking and alcohol consumption among the population, which are known factors affecting the hematological and micronutrient parameters. Another limitation of this study is the absence of diagnostic screening for prevalent subclinical conditions (e.g., asymptomatic hemoglobinopathies and parasitic infestations), which may have introduced residual variability. Nevertheless, questionnaire-based information and the Latent Value Exclusion (LAVE) method were applied to minimize the impact of undiagnosed conditions on the RIs.

## 5. Conclusions

In conclusion, our study provides valuable RIs for CBC and micronutrient parameters, tailored to the urban Dhaka population. Considering demographic factors, applying the LAVE method, and thoroughly analyzing variations enhance the precision and applicability of the derived RIs. Further studies on populations in other urban and rural areas of Bangladesh are needed to generate country-specific RIs. These RIs are essential tools for healthcare professionals, aiding accurate diagnoses, treatment decisions, and public health planning in Bangladesh.

## Figures and Tables

**Table 1:** General characteristics of the study participants.

Items	Overall	Slum, *n* = 1181	Non-Slum, *n* = 543	*p* Value
Age, years	30.0 ± 15.1	30.5 ± 15.4	29.0 ± 14.5	0.057
Age, category				
10–17 years	417 (24.2%)	125 (23.0%)	292 (24.7%)	
18–40 years	921 (53.4%)	307 (56.5%)	614 (52.0%)	
41–60 years	322 (18.7%)	95 (17.5%)	227 (19.2%)	
>60 years	64 (3.7%)	16 (2.9%)	48 (4.1%)	
Sex				
Female	962 (55.8%)	646 (54.7%)	316 (58.2%)	0.175
Male	762 (44.2%)	535 (45.3%)	227 (41.8%)	
Antibody response				
Non-reactive	496 (28.8%)	305 (25.8%)	191 (35.2%)	<0.001
Reactive	1228 (71.2%)	876 (74.2%)	352 (64.8%)	
BMI				
Normal	935 (54.2%)	654 (55.4)	281 (51.7)	
Overweight	525 (30.5%)	318 (26.9)	207 (38.1)	
Underweight	264 (15.3%)	209 (17.7)	55 (10.1)	
Occupation				
Student	464 (26.9%)	272 (23.0%)	192 (35.4%)	
Service	277 (16.1%)	189 (16.0%)	88 (16.2%)	
Business	146 (8.5%)	87 (7.4%)	59 (10.9%)	
Home maker	386 (22.4%)	245 (20.7%)	141 (26.0%)	
Self-employed	190 (11.0%)	170 (14.4%)	20 (3.7%)	
Unemployed	261 (15.1%)	218 (18.5%)	43 (7.9%)	
Family income				
<20,000	680 (39.4%)	669 (56.6%)	11 (2.0%)	
20,000–40,000	556 (32.2%)	439 (37.2%)	117 (21.5%)	
40,000–70,000	343 (19.9%)	73 (6.2%)	270 (49.7%)	
>70,000	145 (8.41%)	0 (0.0%)	145 (26.7%)	

Notes: Data are presented as mean ± SD or number with percent in parentheses.

**Table 2:** Multiple regression analysis for sources of variation in reference values in males and females.

Test Items	Male						Female					
	*n*	R	Age	Reactive *	Slum	BMI	*n*	R	Age	Reactive *	Slum	BMI
VitD, ng/mL	426	0.534	**0.344**	0.024	**0.384**	−0.100	568	0.408	**0.203**	0.002	**0.326**	0.095
Zinc, µg/mL	484	0.343	−0.198	0.162	**0.222**	0.180	666	0.290	−0.132	0.014	**0.255**	−0.022
RBC, 10/µL	758	0.266	**−0.256**	0.004	−0.033	0.162	961	0.235	**−0.240**	−0.013	0.071	0.104
Hb, g/dL	761	0.107	−0.051	−0.023	0.022	0.111	956	0.179	−0.151	−0.030	0.118	0.072
Ht, %	762	0.115	−0.064	0.002	−0.007	0.114	962	0.186	−0.174	0.001	0.098	0.090
MCV, fL	762	0.263	**0.268**	−0.012	0.034	−0.093	962	0.116	0.113	0.007	0.030	−0.034
MCH, pg	762	0.263	**0.258**	−0.037	0.063	−0.082	962	0.090	0.072	−0.001	0.049	−0.035
MCHC, g/dL	762	0.113	0.031	−0.062	0.094	0.015	952	0.094	−0.052	−0.024	0.075	−0.011
RDW-SD, fL	759	0.257	**0.246**	−0.054	0.063	−0.027	958	0.174	0.138	0.072	0.010	0.040
RDW-CV, fL	761	0.079	−0.022	−0.032	0.025	0.078	958	0.079	0.034	0.027	−0.007	0.055
WBC, k/µL	761	0.143	−0.037	−0.074	0.108	0.097	960	0.232	−0.194	−0.029	0.109	0.178
Neu, %	762	0.228	**0.194**	−0.004	−0.073	0.053	962	0.168	0.075	−0.029	−0.111	0.073
Lym, %	762	0.213	**−0.209**	−0.002	0.046	−0.001	962	0.091	−0.054	0.008	0.067	−0.019
Mon, %	762	0.083	0.014	−0.058	−0.052	−0.002	962	0.197	−0.010	−0.036	−0.115	−0.160
Eos, %	761	0.168	−0.049	0.039	0.077	−0.110	961	0.246	−0.071	0.064	0.174	−0.112
Bas, %	760	0.077	0.035	0.057	0.026	0.018	961	0.148	0.092	0.056	0.092	−0.026
PLT, k/µL	759	0.132	−0.113	−0.061	0.032	0.088	960	0.129	−0.065	−0.036	0.001	0.128
MPV, fL	733	0.207	−0.083	0.051	−0.160	0.091	908	0.202	0.085	−0.018	**−0.192**	−0.050
P-LCR, %	733	0.222	−0.096	0.057	−0.165	0.104	908	0.216	0.087	−0.014	**−0.208**	−0.046
PCT, %	733	0.196	−0.166	−0.065	−0.039	0.127	908	0.147	−0.041	−0.063	−0.069	0.107
PDW, fL	676	0.251	−0.135	0.038	−0.150	0.158	819	0.242	0.092	−0.019	**−0.233**	−0.041

Notes: Standardized partial regression coefficients (r_p_) are listed in the table with |r_p_| ≥ 0.20 marked in bold letters. R: multiple correlation coefficient; Reactive *: positive anti-SARS-CoV-2 antibody.

**Table 3:** Standard deviation ratio of hematology markers and micronutrients for age and sex.

Markers	SDRsex	SDRage	SDRage M	SDRage F
VitD, ng/mL	*0.435*	**0.379**	**0.390**	**0.364**
Zinc, µg/mL	0.308	0.209	0.256	0.149
RBC, 10/µL	*1.112*	0.265	0.335	0.146
Hb, g/dL	*1.266*	0.346	*0.456*	0.165
Ht, %	*1.218*	0.347	*0.471*	0.140
MCV, fL	0.079	0.217	0.299	0.091
MCH, pg	0.059	0.265	**0.389**	0.080
MCHC, g/dL	*0.468*	0.000	0.000	0.000
RDW-SD, fL	0.000	0.219	0.280	0.132
RDW-CV, fL	0.079	0.187	0.226	0.143
WBC, k/µL	0.148	0.209	0.285	0.106
Neu, %	0.000	0.168	0.211	0.119
Lym, %	0.292	0.095	0.000	0.160
Mon, %	0.195	0.178	0.219	0.117
Eos, %	0.183	0.000	0.000	0.120
Bas, %	0.216	0.127	0.224	0.000
PLT, k/µL	0.097	0.102	0.174	0.000
MPV, fL	0.096	0.106	0.178	0.000
P-LCR, %	0.324	0.200	0.298	0.086
PCT, %	0.042	0.106	0.190	0.000

Notes: SDR: standard deviation ratio, M: male, F: female. SDRs ≥ 0.35 are shown in bold; SDRs ≥ 0.40 are shown in italic.

**Table 4:** Bangladeshi RIs for micronutrient parameters and CBC established parametrically with the application of the LAVE procedure.

Items	Male + Female		Male		Female	
	*N*	LL–UL	*N*	LL–UL	*N*	LL–UL
VitD, ng/mL	698	**4.03–27.5**	301	**4.82–30.7**	397	**4.02–24.2**
Zinc, µg/mL	813	0.55–1.05	352	0.56–1.10	470	0.55–1.00
RBC, 10/µL	1225	**40** **4–** **602**	541	**44** **3–** **60** **5**	668	**38** **8–** **52** **4**
Hb, g/dL	1259	**10.7–16.6**	558	**12.1–16.7**	693	**10.2–14.4**
Ht, %	1227	**35.1–50.7**	542	**38.4–50.6**	669	**33.6–44.1**
MCV, fL	1228	75.4–93.9	542	75.9–93.8	669	75.0–93.9
MCH, pg	1265	23.4–30.6	556	23.8–30.8	687	23.0–30.4
MCHC, g/dL	1262	**30.3–34.5**	563	**30.9–34.9**	694	**29.9–33.9**
RDW-SD, fL	1226	35.5–44.6	542	35.4–44.7	669	35.6–44.8
RDW-CV, fL	1265	11.7–15.0	**562**	**11.7–14.7**	**691**	**11.7–15.4**
WBC, k/µL	1272	4.93–11.8	562	4.74–11.2	692	4.92–12.2
Neu, %	1271	40.4–73.7	561	38.8–72.4	691	42.4–74.8
Lym, %	1228	21.5–48.6	541	22.1–49.5	669	21.6–47.7
Mon, %	1224	1.95–4.97	**541**	**2.17–5.27**	**665**	**1.83–4.62**
Eos, %	1215	1.12–13.7	**537**	**1.45–15.6**	**664**	**0.87–11.8**
Bas, %	1227	0.02–0.34	542	0.01–0.36	669	0.01–0.31
PLT, k/µL	1284	103–438	569	**97.2–412**	693	**113–45** **1**
MPV, fL	1197	9.23–13.4	532	9.19–13.2	646	9.30–13.5
P-LCR, %	1199	17.2–51.7	533	17.3–50.7	643	18.0–52.7
PCT, %	1198	0.16–0.44	531	0.17–0.41	648	0.17–0.46

Notes: LL: lower limit, UL: upper limit. The values in bold font represents RIs adopted considering sex.

## Data Availability

All data underlying the findings in our study are freely available in the manuscript and Supplementary Materials. For additional information, please refer to http://www.icddrb.org/policies (accessed on 21 June 2026).

## Supplementary Materials

The following supporting information can be downloaded at: https://www.mdpi.com/article/10.3390/biom16070968/s1, Table S1: Determination of RIs for CBC and micronutrients (Vitamin D and Zinc) utilizing a parametric method, and bias assessment employing the LAVE method; Table S2: Determination of RIs for CBC and micronutrients (Vitamin D and Zinc) utilizing a non-parametric method before and after latent abnormal values exclusion; Table S3: Differences between global Reference Intervals (RIs) and Bangladeshi ranges reported in this study.

## Abbreviations

The following abbreviations are used in this manuscript:

BMI	Body mass index
F	Female
M	Male
RBCs	Red blood cells
Hb	Hemoglobin
Ht	Hematocrit
MCV	Mean corpuscular volume
MCH	Mean corpuscular hemoglobin
MCHC	Mean corpuscular hemoglobin concentration
RDW-SD	Red cell distribution width—standard deviation
RDW-CV	Red cell distribution width—coefficient of variation
WBCs	White blood cells
Neu	Neutrophil
Lym	Lymphocyte
Mon	Monocyte
Eos	Eosinophil
Bas	Basophil
PLT	Platelet
MPV	Mean platelet volume
P-LCR	Platelet large cell ratio
PCT	Plateletcrit
PDW	Platelet distribution width
VitD	Vitamin D
mL	Milliliter
µL	Microliter
dL	Deciliter
fL	Femtoliter
ng	Nanogram
µg	Microgram
pg	Picogram
k/µL	Thousands per microliter
