# Supplementary material for: Defining Reference Intervals for Complete Blood Count and Micronutrient Parameters in Urban Bangladeshi Population
Source: Biomolecules. 2026 Jun 30;16(7):968. doi: 10.3390/biom16070968 (PMC13406479; doi:10.3390/biom16070968)
Supplement: Supplementary file 1 [file biomolecules-16-00968-s001.zip › Table S2.pdf]

Table S2. RIs for CBC and micronutrients (Vitamin D and Zinc) calculated by non-parametric method before and after latent abnormal values exclusion.

Test Items		Male + Female				Male				Female			
	LAVE	N	LL	Me	UL	N	LL	Me	UL	N	LL	Me	UL
Vitamin D	(-)	996	4.18	12.5	27.9	427	4.92	15.0	31.7	569	3.85	11.1	23.4
	(+)	699	4.09	12.9	27.7	302	4.84	15.7	30.5	397	3.74	11.3	23.8
Zinc	(-)	1150	0.54	0.76	1.03	484	0.55	0.80	1.07	666	0.53	0.74	1.00
	(+)	814	0.55	0.76	1.04	352	0.57	0.80	1.09	471	0.55	0.74	0.98
RBC	(-)	1724	380.7	480.5	612.4	762	417.9	523.0	637.2	962	372.3	454.5	556.0
	(+)	1228	400.4	481.4	590.9	542	440.8	522.5	604.1	669	384.0	453.7	524.5
Hb	(-)	1724	9.43	13.0	16.2	762	11.1	14.3	16.6	962	8.94	12.19	14.77
	(+)	1262	10.5	13.3	16.2	559	12.0	14.5	16.6	696	10.1	12.3	14.5
Hematocrit	(-)	1724	31.2	40.5	49.6	762	35.1	44.1	50.4	962	29.8	38.4	45.3
	(+)	1228	35.0	41.2	49.3	542	38.6	44.5	50.3	669	33.4	38.7	44.0
MCV	(-)	1724	66.1	85.3	94.4	762	67.2	85.1	94.8	962	66.1	85.5	94.2
	(+)	1228	74.4	86.0	93.7	542	75.0	85.5	93.6	669	73.6	86.1	93.8
MCH	(-)	1724	20.0	27.5	30.6	762	21.4	27.8	30.8	962	19.3	27.2	30.4
	(+)	1268	23.2	27.7	30.6	558	23.6	28.0	30.8	688	23.0	27.3	30.3
MCHC	(-)	1724	29.4	32.0	34.6	762	30.5	32.5	34.9	962	28.9	31.6	34.3
	(+)	1263	30.3	32.1	34.5	565	30.7	32.5	34.8	694	29.9	31.7	34.0
RDW-SD	(-)	1723	34.8	39.6	46.3	762	34.7	39.4	46.3	961	34.8	39.7	46.2
	(+)	1228	35.4	39.5	45.0	542	35.2	39.5	45.0	669	35.5	39.5	45.0
RDW-CV	(-)	1723	11.7	13.0	17.7	762	11.8	13.0	17.0	961	11.6	13.0	18.0
	(+)	1276	11.7	12.9	15.3	566	11.7	12.9	15.0	697	11.7	12.9	15.5
WBC	(-)	1723	4.74	7.75	12.3	762	4.72	7.61	12.5	962	4.81	7.95	12.0
	(+)	1276	4.83	7.70	11.8	564	4.68	7.50	11.6	693	4.91	7.96	12.0
Neutrophils%	(-)	1724	40.6	58.1	75.6	762	38.5	56.8	73.3	962	42.3	59.0	76.6
	(+)	1273	40.3	57.8	74.0	562	37.7	56.7	72.0	693	42.4	59.1	75.5
Lymphocyte%	(-)	1724	19.0	33.6	49.6	762	19.3	34.3	50.8	962	18.6	33.1	48.6
	(+)	1228	21.6	34.0	48.2	542	22.1	34.2	48.9	669	21.6	33.5	46.8
Monocyte%	(-)	1724	1.81	3.10	5.03	762	1.98	3.28	5.21	962	1.74	2.98	4.80
	(+)	1228	1.93	3.12	5.01	542	2.19	3.29	5.18	669	1.81	2.99	4.77
Eosinophils%	(-)	1722	0.83	3.93	14.6	761	1.11	4.33	15.1	961	0.72	3.58	13.4
	(+)	1226	0.97	3.95	14.6	542	1.28	4.37	15.1	668	0.79	3.58	12.7
Basophils%	(-)	1724	0.00	0.10	0.30	762	0.00	0.11	0.33	962	0.00	0.10	0.30
	(+)	1228	0.00	0.10	0.30	542	0.00	0.11	0.31	669	0.00	0.10	0.30
PLT	(-)	1719	75.3	280.0	454.7	759	76.3	262.3	434.8	960	73.8	293.3	466.4
	(+)	1286	82.3	278.0	449.9	570	73.2	258.6	424.8	694	97.4	292.4	453.2
MPV	(-)	1641	9.21	10.7	13.2	733	9.24	10.6	13.1	908	9.20	10.7	13.2
	(+)	1199	9.23	10.7	13.2	533	9.20	10.7	13.3	648	9.21	10.7	13.3
P_LCR	(-)	1641	17.5	30.1	50.6	733	17.3	29.6	49.1	908	17.6	30.7	51.0
	(+)	1199	17.4	30.1	50.7	533	17.4	30.0	50.3	648	17.3	30.4	51.3
PCT	(-)	1641	0.08	0.31	0.47	733	0.09	0.29	0.44	908	0.08	0.33	0.48

	(+)	1199	0.15	0.30	0.43	533	0.14	0.28	0.40	648	0.16	0.32	0.46
PDW	(-)	1495	9.03	11.8	18.2	676	8.89	11.7	18.1	819	9.13	12.0	18.4
	(+)	1088	9.08	11.8	18.4	481	9.13	11.8	18.4	579	9.19	11.9	18.5

Note: LL: lower limit, Me: median, UL: upper limit, LAVE: latent abnormal values exclusion, (-): without LAVE, (+): with LAVE.

VitD: vitamin D, RBC: red blood cells, Hb: hemoglobin, Ht: hematocrit, MCV: mean corpuscular volume, MCH: mean corpuscular hemoglobin, MCHC: mean corpuscular hemoglobin concentration, RDW_SD: red cell distribution width-standard deviation, RDW_CV: red cell distribution width - coefficient of variation, WBC-white blood cells, Neu%: neutrophils %, Lym%: lymphocytes %, Mon%: monocytes %, Eos%: eosinophils %, Bas%: basophils %, PLT: platelet count, MPV: mean platelet volume, P_LCR: platelet large cell ratio, PCT: platelet, PWD: platelet distribution width.
